# Supplementary material for: Contrasting evolution of the Arabian Sea and Pacific Ocean oxygen minimum zones during the Miocene
Source: Commun Earth Environ. 2026 Jan 16;7(1):47. doi: 10.1038/s43247-025-03112-4 (PMC12811131; doi:10.1038/s43247-025-03112-4)
Supplement: Supplementary file 2 — Supplementary Material [file 43247_2025_3112_MOESM2_ESM.pdf]

Supplementary Information for

Contrasting evolution of the Arabian Sea and Pacific Ocean oxygen minimum zones during the Miocene

Anya V. Hess and Alexandra Auderset et al.

*Corresponding authors. Email: dranyahess@gmail.com and a.auderset@soton.ac.uk

This PDF file includes:

Supplementary Discussion 1–3
Supplementary Figure 1-9
Supplementary References

Supplementary Discussion 1: Multi-proxy approach to reconstruct ocean oxygenation

I/Ca

The I/Ca proxy relies on the fact that iodate converts to iodide in low-oxygen settings such as ODZs, and iodate can be incorporated into foraminiferal carbonate material whereas iodide cannot (1). Although foraminifera for the most part live above ODZs rather than inside them (e.g., ref. [2]), the iodate signal reflect underlying ODZ oxygenation because iodide oxidation is relatively slow (months to years), so the signal is retained in waters mixing upwards from the ODZ (3). As ODZs are also areas with high productivity, the surface-water iodate concentrations are also partially depleted by iodate reduction by phytoplankton (4). Thus, planktonic foraminifera record lower I/Ca values in ODZs, and I/Ca has been shown to correlate with the minimum oxygen concentration in the water column ($[O_2]_{min}$) (5–7).

In the eastern tropical Pacific ODZ, ref. (7) found distinct patterns of I/Ca values in species that calcify at different depths in areas they term "ODZ core" and "distal ODZ," resulting from patterns in both the strength and depth of the ODZ. However, the Arabian Sea ODZ has a different structure, and as we present data from only surface-calcifying species, we do not attempt to discern those patterns here. Please note that our use of similar terminology herein is not meant to imply the interpretation of the ref. (7) designations.

Mn/Ca

Bulk sediment Mn/Ca ratios are widely used to track bottom-water oxygenation (e.g., refs. (8,9)). In oxygenated settings, manganese is primarily in the form of insoluble Mn^{3+} and Mn^{4+}, and it is reduced to soluble Mn^{2+} in anoxic settings (10). In areas with oxic bottom waters and reducing sediments, Mn oxyhydroxides precipitate in sediments above the subsurface oxycline, enriching the sediments in Mn. With progressive burial, those sediments move below the oxycline, where the Mn oxyhydroxides dissolve. Pore waters traveling upwards that re-encounters the oxycline result in reprecipitation of Mn oxyhydroxides, further concentrating Mn in surface sediments. This process can result in supersaturation of Mn^{2+} in pore waters and, in the presence of calcareous sediments, where dissolution increases the carbonate ion concentration in pore water, this process can result in supersaturation of Mn^{2+} in pore waters and lead to precipitation of Mn-carbonates (11). This is recorded as elevated Mn/Ca ratios measured from gently cleaned foraminifera (12). In contrast, where bottom waters are anoxic, Mn^{2+} diffuses upwards from sediments and escapes into bottom waters, preventing supersaturation. It is also precluded by low export productivity, which results in a deep sedimentary oxycline and prevents Mn from concentrating to such a degree (10,11). Therefore, higher Mn/Ca ratios indicate oxic bottom waters and lower Mn/Ca indicates deoxygenation.

Nitrogen Isotopes

The ratio of nitrogen (N) isotopes ($\delta^{15}N = (^{15}N/^{14}N)_{sample}/(^{15}N/^{14}N)_{atm}N_2 - 1] \times 1000‰$) in the ocean is highly sensitive to low oxygen levels (suboxia, O_2 concentrations <below 5 umol/kg). In suboxic conditions in the water column, denitrification, the bacterial conversion of nitrate to N_2O, preferentially removes isotopically light ^{14}N, leaving the residual seawater nitrate enriched in isotopically heavy ^{15}N. This process leads to elevated $\delta^{15}N$ of nitrate in the ODZ (13,14) and depending on the extent of this water column process it can contribute to the elevation of the mean ocean nitrate $\delta^{15}N$ in the global ocean, which is driven by the relative contributions of water column versus sedimentary denitrification (14,15).

The analysis of nitrogen isotopes on the intracrystalline organic matter of planktic foraminifera (foraminifera-bound nitrogen isotopes) can be used to reconstruct the geologic history of water column denitrification and thus oxygenation history over millions of years (16–19). In contrast to the analysis of

organic matter in bulk sediments, the calcite-bound organic matter is less prone to degradation and secondary overprints in the water column or sediment and seems to be less affected by diagenesis (20). In areas of complete nutrient consumption, the FB-$\delta^{15}N$ of symbiont-bearing foraminifera reflect the $\delta^{15}N$ of subsurface nitrate (~0–200m), which comprises a combination of the mean ocean nitrate $\delta^{15}N$ signal and signatures of the regional processes (nitrogen fixation, water column denitrification, or sedimentary denitrification) (13,21,22).

Supplementary Discussion 2: Diagenetic influence on trace elements

I/Ca

Since authigenic minerals are present, we must evaluate whether the I/Ca and Mg/Ca trace elemental ratios reflect a primary signal acquired at the ocean surface or secondary processes associated with diagenetic carbonate overgrowth on the shell. Authigenic carbonates (e.g., $MnCO_3$) have elevated Mn/Ca, Mg/Ca, and in some cases also Fe/Ca (23,24). Precipitated at the redox boundary where iodine exists primarily as iodide (I^-), they are expected to have lower [IO_3^-] and therefore lower I/Ca ratios (25). However, removal of Mn-oxides by reductive cleaning has been shown to either have no effect on I/Ca values (26) or to lower them (27), so it is unlikely that the Site 714 I/Ca values are lowered by the presence of Mn-carbonates in the absence of reductive cleaning. Mn-Fe oxides have elevated Mn/Ca, Mg/Ca, and Fe/Ca (24). As they are not associated with elevated [Ca] (24) and we see no reason why they would contain iodine, we expect that they would not significantly impact I/Ca ratios. Since Mg/Ca values at Site 714 are low even during the warm MCO, Mg/Ca appears to be unaffected and the effect on I/Ca may be minor. Despite the apparent presence of diagenetic carbonates in Site 714 samples indicated by elevated Mn/Ca ratios, similar I/Ca values here and in Site 730 samples, which underwent reductive cleaning and do not have elevated Mn/Ca ratios, suggests that the I/Ca signal is primary.

Sr/Ca

Sr/Ca ratios are also lower in diagenetically altered foraminifera because Sr^{2+} is released during dissolution and not incorporated into diagenetic carbonate (28). At Site 730, Sr/Ca ratios are high (~1.61±0.07 mmol/mol), suggesting little diagenetic alteration (Fig. S3d). At Site 714, values rise from ~1.16±0.04 mmol/mol in samples >16 Ma to ~1.26±0.02 mmol/mol in samples <13.5 Ma. These values and this trend are similar to those at ETP Site 845, where I/Ca ratios are primary and are relatively high >~15 Ma (18). We posit that Sr/Ca values at Site 714 are primary, perhaps responding to the increasing pH through the Miocene documented by ref. (29), suggesting that I/Ca values may also be primary, as at Site 845.

Mn/Ca

Recent work has shown that the primary Mn/Ca signal in planktonic foraminiferal calcite increases under upwelling conditions (30) and decreases with increasing water-column oxygen concentrations (31). However, Mn/Ca ratios in primary calcite are typically much lower (i.e., <~30–100 umol/mol) than in authigenic carbonate (>~200–1000 umol/mol) (23,30,31) and in planktonic foraminifera with authigenic carbonate ($MnCO_3$), the Mn/Ca signal is dominated by that of the authigenic carbonate (23). When planktonic foraminiferal samples are not reductively cleaned, authigenic Mn-carbonate coatings precipitated in the sediment are less likely to have been removed (23,32). Since samples from Site 714 were not reductively cleaned, it is likely that some of the authigenic carbonate observed in those samples

in SEM remained on the foraminiferal samples, and we use their Mn/Ca ratios, dominated by authigenic $MnCO_3$ overgrowth, to infer bottom water oxygenation.

Supplementary Discussion 3: Proxies respond to progressively lower oxygen concentrations

Manganese responds at the highest oxygen concentrations, followed by iodine, owing to the difference in their reduction potentials (E°=1.29 volts for manganese dioxide, E°=1.23 volts for iodate). Ref. (33) observed a maximum in dissolved manganese at <100 µmol/kg oxygen concentrations. Mn/Ca is typically used to interpret relative rather than quantitative changes in oxygenation (e.g., (8,9)). We do not compare absolute values of Mn/Ca because the proxy is qualitative and may have different values in bulk sediments and foraminiferal overgrowths. The oxygen concentration at which iodate converts to iodide remains unknown, with values of <70–100 or as low as <1 µmol/kg suggested (6,34). I/Ca in planktonic foraminifera varies with the minimum oxygen concentration in the water column, with the lowest I/Ca values in the core of OMZs, where $[O_2]_{min}$ is <10 µmol/kg (7). Finally, based on previous studies in the Arabian Sea, water column denitrification is described when bulk sedimentary $\delta^{15}N$ values exceed 6 ‰ (9,35–38). However, this threshold could be different for bulk sedimentary $\delta^{15}N$ and FB-$\delta^{15}N$. For example, we observe an average offset between $\delta^{15}N_{bulk}$ and FB-$\delta^{15}N$ of ~1.70±0.50 ‰ in Site 714 (Figs. S4c, S9). Therefore, the FB-$\delta^{15}N$ denitrification threshold would be expected to be around 7.7‰. In comparison, the overall elevated FB-$\delta^{15}N$ values from ETNP Site 845 during the MCO may be due to a species-offset effect (39,40), as the record in Site 845 was measured on the symbiont-barren *Dentoglobigerina venezuelana* and the records in the Arabian Sea were measured on dinoflagellate symbiont-bearing *Trilobatus sacculifer*. Thus indicating that a denitrification threshold for FB-$\delta^{15}N$ is highly uncertain and might vary depending on ocean region and sediment material/ foraminiferal species (41). Instead of interpreting FB-$\delta^{15}N$ thresholds, we focus on general trends for this study.

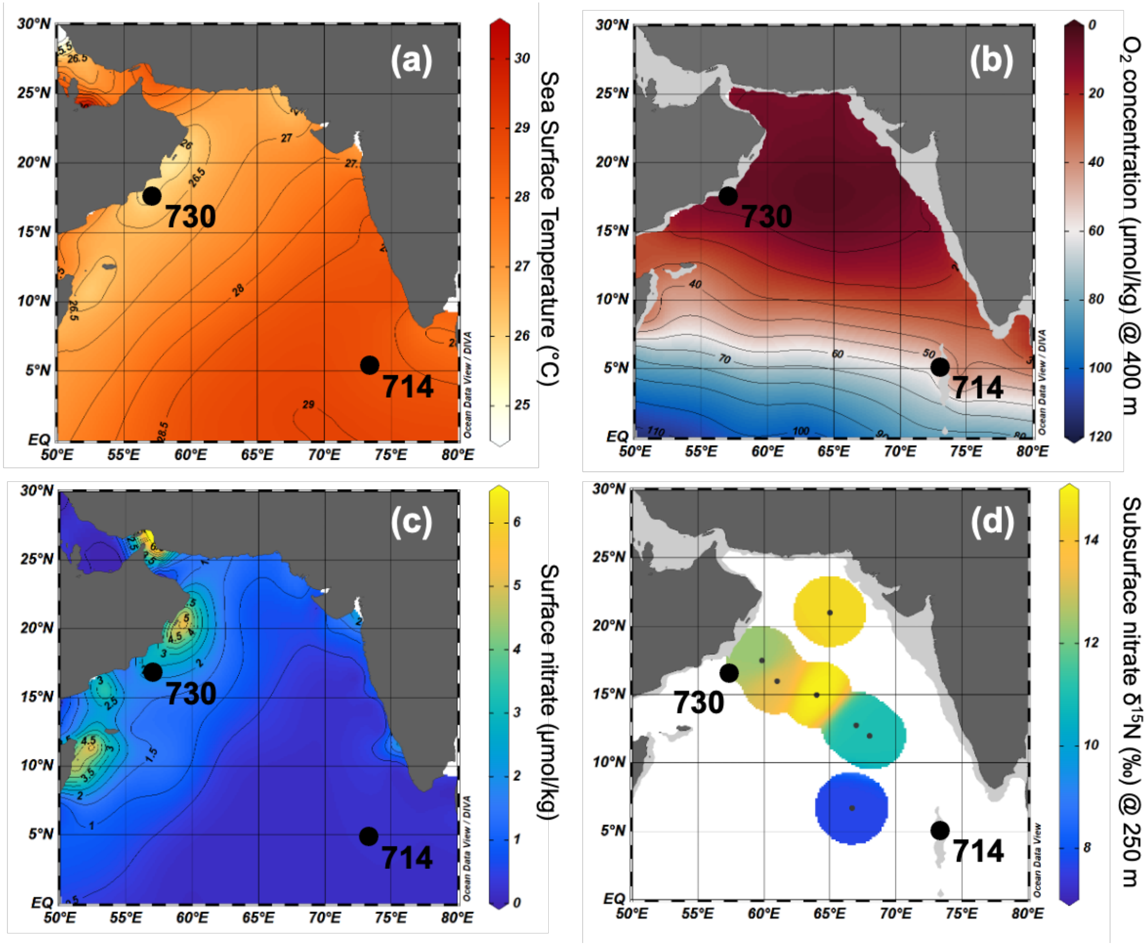

Supplementary Figure 1. Map of the Arabian Sea showing **(a)** mean annual sea surface temperatures (42), **(b)** oxygen concentration at 400 m water depth (43), **(c)** surface nitrate concentrations (in µmol/kg) (43) **(d)** subsurface nitrate $\delta^{15}N$ at 250 m water depth (44). Generated in Ocean Data View v. 5.7.1 (45) by DIVA gridding 50x50 grid cells, signal-to-noise ratio of 50.

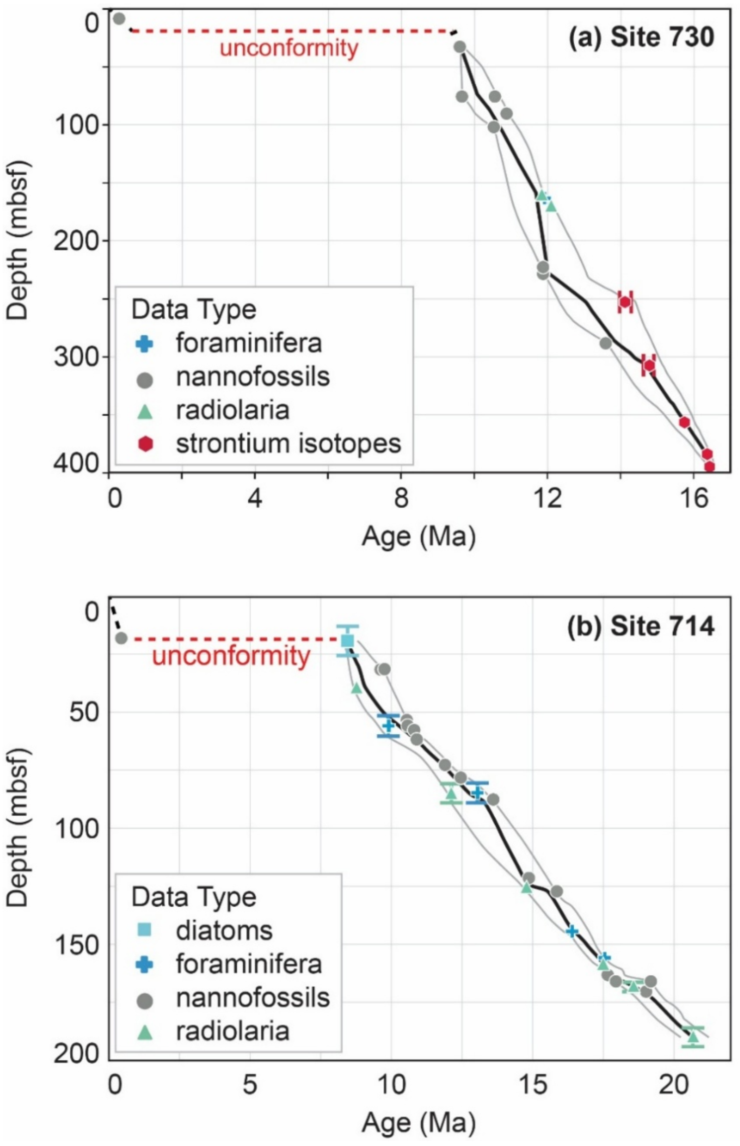

Supplementary Figure 2. Age-depth correlation for Arabian Sea sites. Age-depth correlations (black lines) and 95% confidence intervals (gray lines) from Undatable (46) run with xfactor of 0.1 and bootstrapping 30%. All ages updated to ref. (47) (Supplementary Data 1). Unless shown, age and depth error bars are smaller than symbols. (a) Site 730. Biostratigraphic depths from ref. (48). **(b)** Site 714. Biostratigraphic depths from ref. (49). Strontium isotope data from ref. (50).

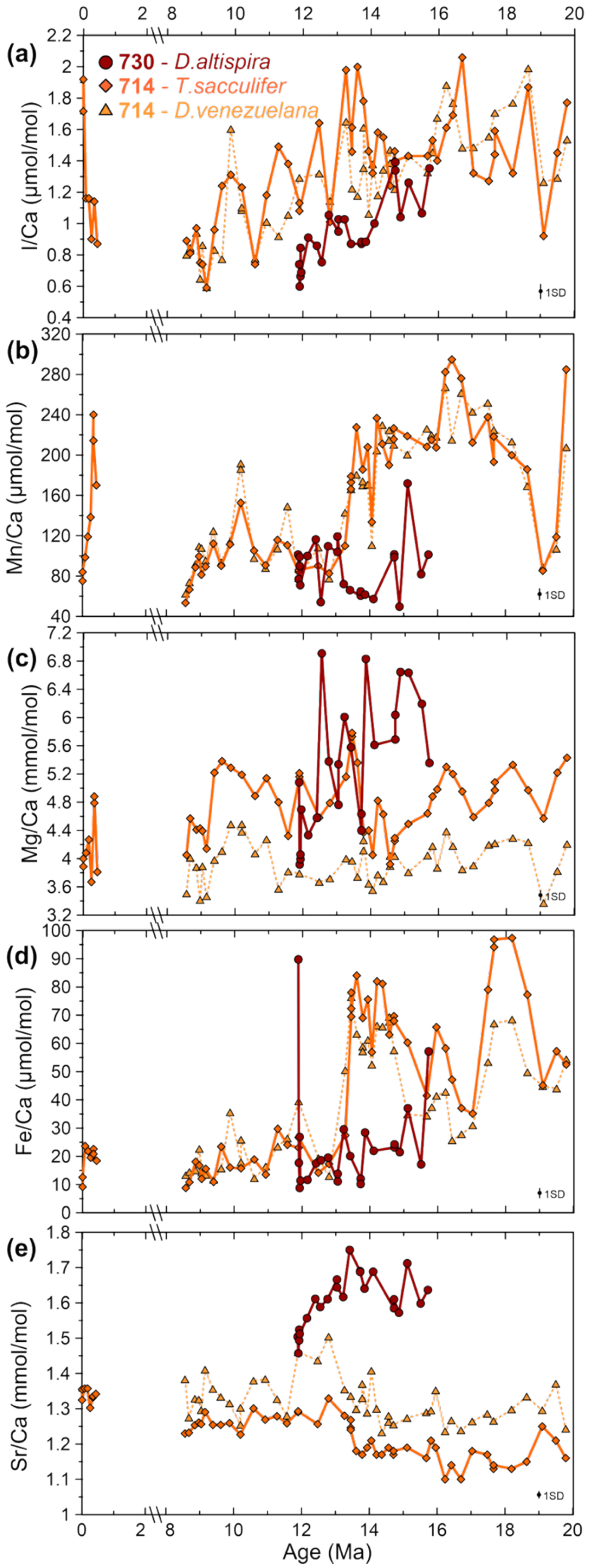

Supplementary Figure 3. Trace elemental data from ODP Sites 714 and 730 in surface dwellers *T. sacculifer* and *Dentoglobigerina altispira* and subsurface dweller *Dentoglobigerina venezuelana*. **(a)** I/Ca. **(b)** Mn/Ca. **(c)** Mg/Ca. **(d)** Fe/Ca. **(e)** Sr/Ca. Error bars indicate 1 sigma standard deviation.

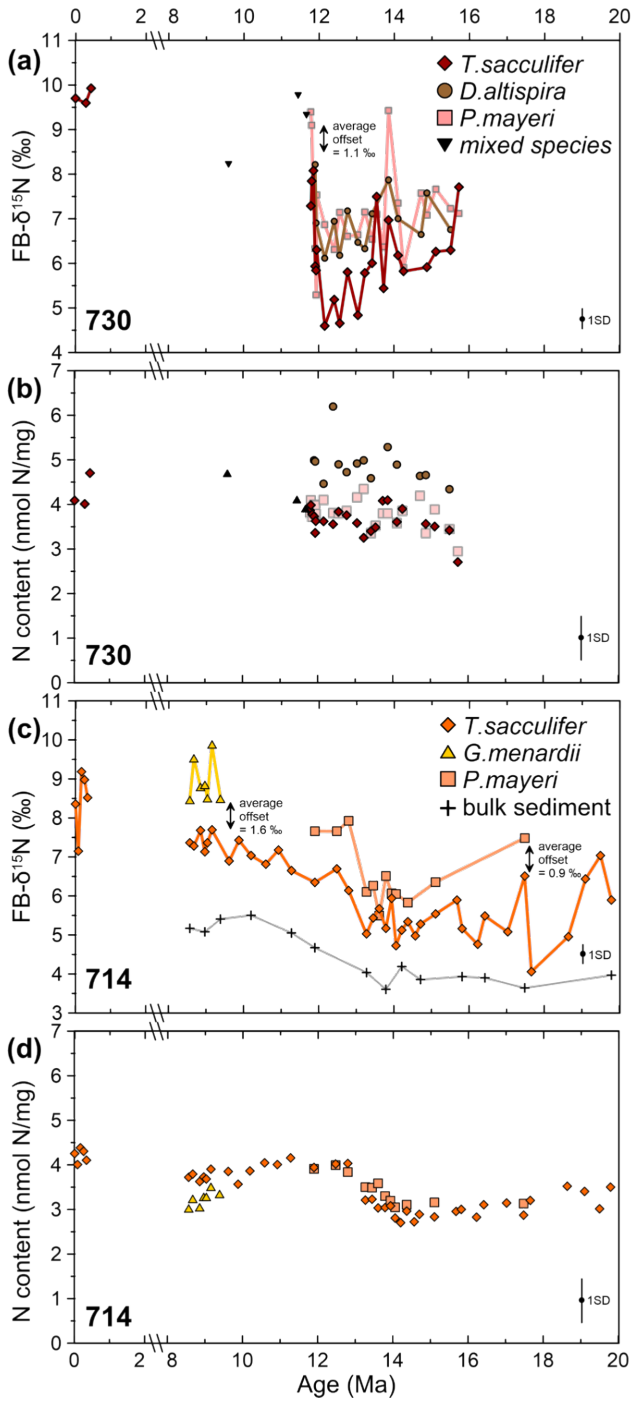

Supplementary Figure 4. Nitrogen isotope data from ODP Sites 714 and 730 in surface and subsurface-dwellers. **(a)** FB-δ^{15}N in Site 714. Arrows indicate average species offset between *T. sacculifer* and *Globorotalia menardii* and *Paragloborotalia mayeri*, respectively. **(b)** N content in Site 714 for *T. sacculifer*, *G. menardii*, and *P. mayeri*. **(c)** FB-δ^{15}N in Site 730. Arrow indicates average species/genus offset between *T. sacculifer* and Dentoglobigerina mixed species. Open diamonds indicate offset-corrected *T. sacculifer* data derived from Dentoglobigerina mixed species. **(d)** N content in Site 730. Error bars indicate 1 sigma standard deviation.

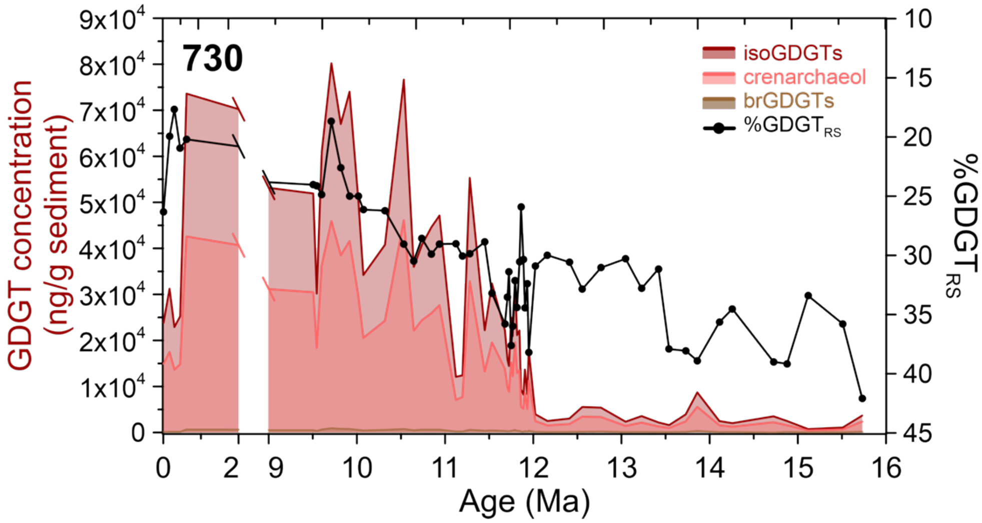

Supplementary Figure 5. GDGT concentrations and Red Sea-type influence on archaeal distribution for Site 730. Isoprenoid GDGTs in dark red, Crenarchaeol in pink and branched GDGTs in brown. Concentrations in ng/g sediment (read values from left-hand y-axis). $\%GDGT_{RS}$ after (51)) in black (read values from the right-hand y-axis). Note the right-hand y-axis is reversed.

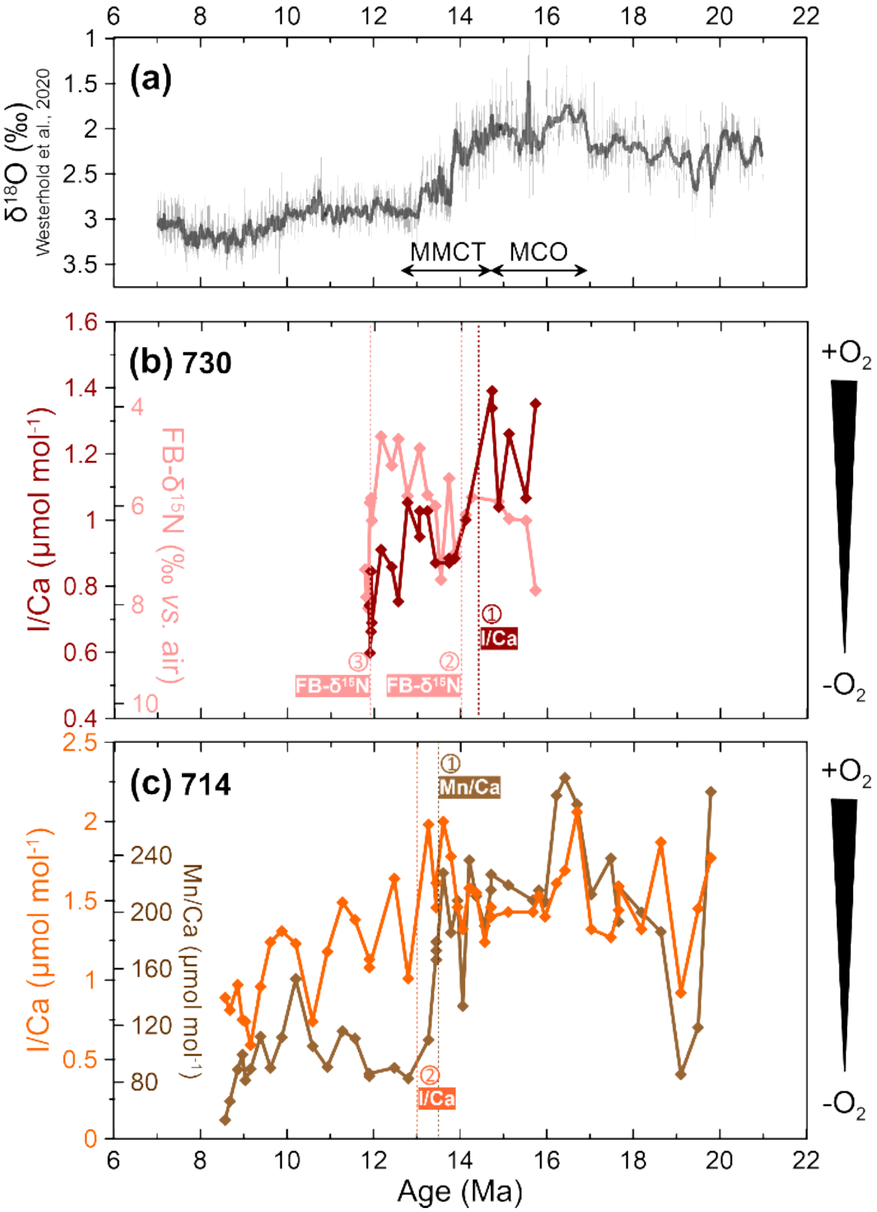

Supplementary Figure 6. Oxygenation proxies by site showing deoxygenation beginning following the MCO. **(a)** oxygen isotopes from benthic foraminifera (52), for climatic context. **(b)** I/Ca and $\delta^{15}N$ measured on *T. sacculifer* at Site 730 in core of modern ODZ. **(c)** I/Ca, Mn/Ca and $\delta^{15}N$ measured on *T. sacculifer* at Site 714 in distal ODZ. Dashed lines with numbers show shifts in oxygenation proxies. Note that Mn/Ca shifts first, followed by I/Ca, then $\delta^{15}N$, responding to progressively lower oxygen concentrations. Also note that each proxy responds first at Site 730 then later at Site 714. FB-$\delta^{15}N$ axes are flipped compared to in Figure 2.

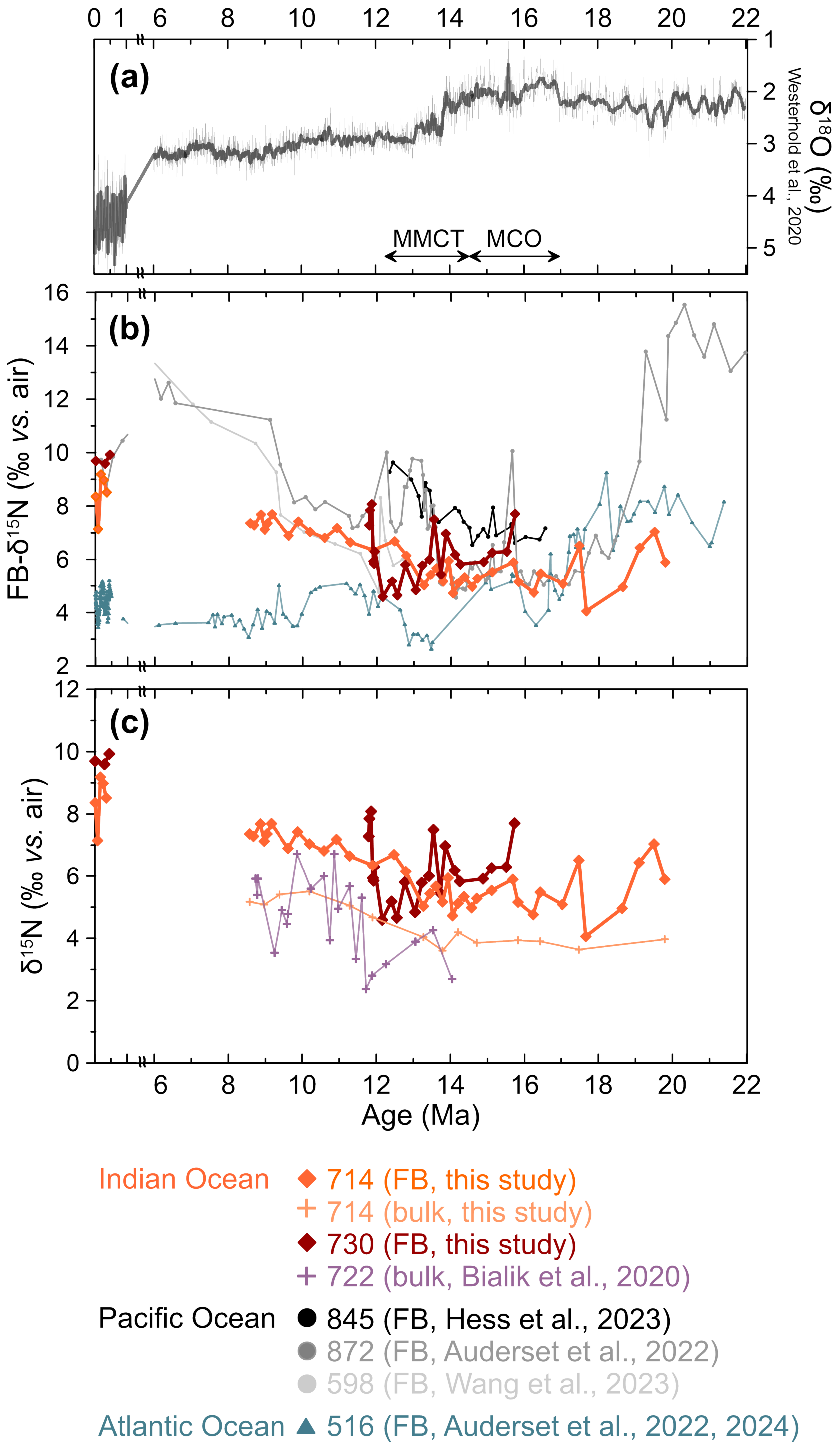

Supplementary Figure 7. Comparison of global and regional foraminifera-bound (FB) and bulk sediment N-isotope records **(a)** Oxygen isotopes from benthic foraminifera (52), **(b)** FB-$\delta^{15}N$ data from symbiont-bearing planktic foraminifera in the Pacific Ocean (ODP 872 (17), ODP 845 (18)), ODP 598 (mixed-species only) (53), Atlantic Ocean (DSDP 516 (17,54)) and Arabian Sea (ODP 730 and ODP 714, this study). **(c)** $\delta^{15}N$ measured on *T. sacculifer* in Sites 714 and 730 and bulk sediment in Site 714 (this study) and Site 722 (9).

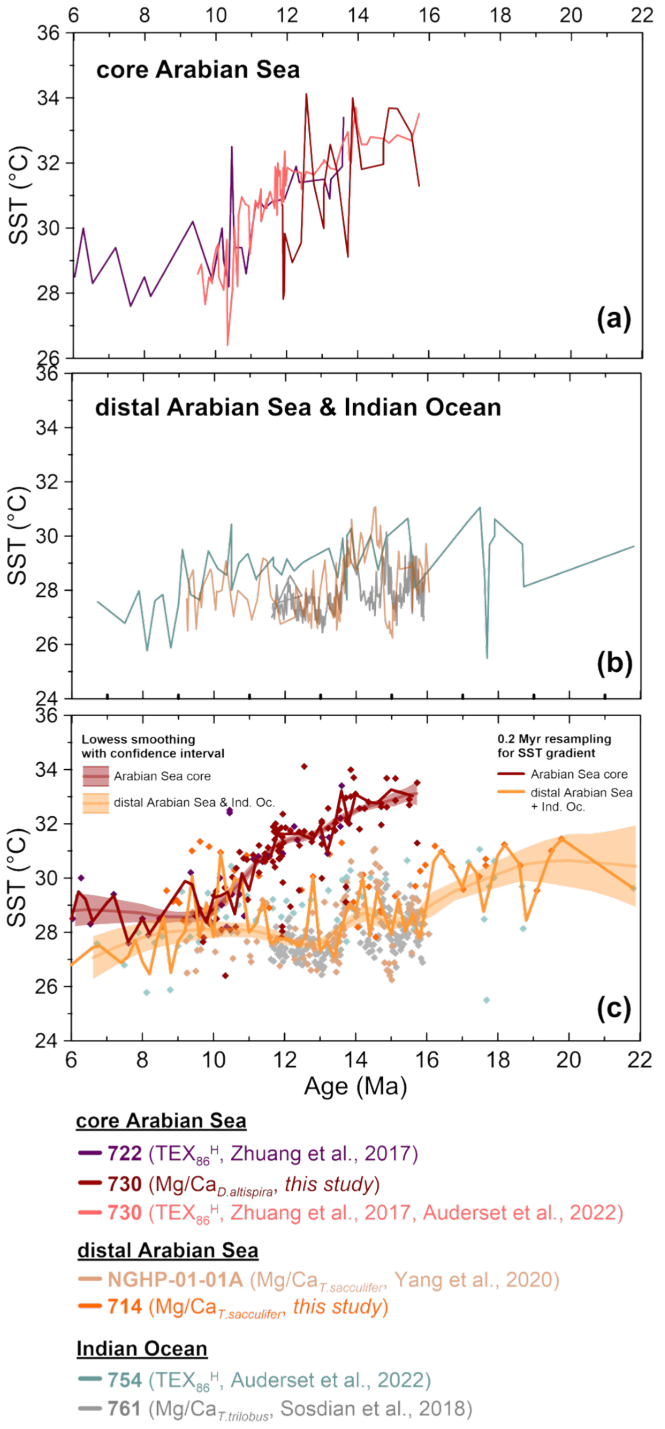

Supplementary Figure 8. Sea surface temperature (SST) compilation with core sites from the Arabian Sea (core and distal) and open Indian Ocean. **(a)** Sea surface temperature reconstructions for the core of the Arabian Sea in Site 730 (Mg/Ca, this study and TEX_{86}^{H} (17,55)) and Site 722 (TEX_{86}^{H} (55)). **(b)** SST for distal Arabian Sea sites NGHP (Mg/Ca (56)) and 714 (Mg/Ca, this study). Open Indian Ocean sites include Site 754 (TEX_{86}^{H} (17)) and Site 761 (Mg/Ca (29)). **(c)** Lowess smoothing with confidence interval for core Arabian Sea sites (light red line with envelope) and distal Arabian Sea sites combined with open Indian Ocean sites (light orange line with envelope). Red and orange lines indicate 0.2 Myr resampled combined SST records which is used for the SST gradient in Figure 3b.

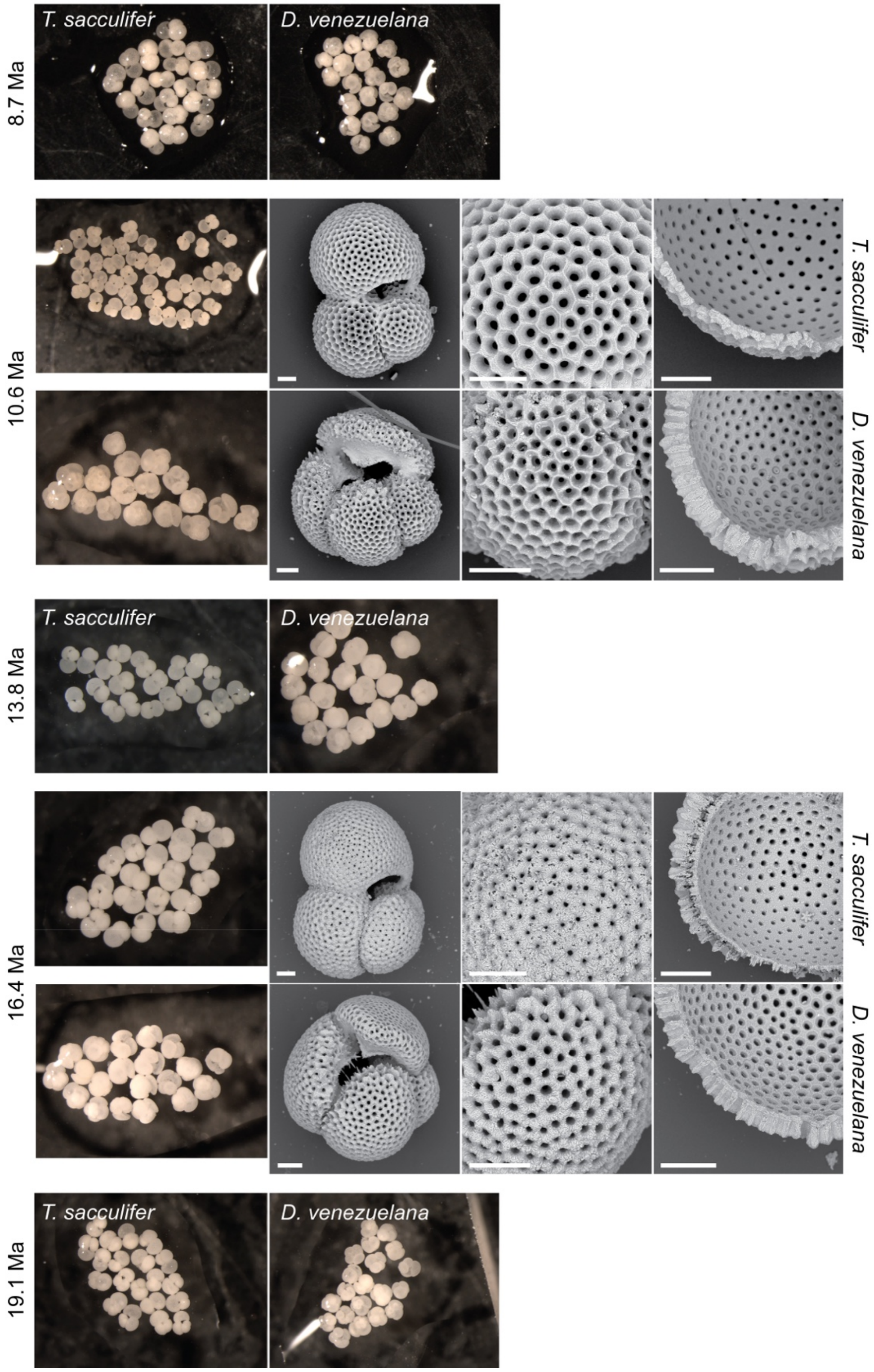

Supplementary Figure 9. Foraminifera preservation. Light microscope and scanning electron microscope images of foraminifera *T. sacculifer* and *Dentoglobigerina venezuelana* from Site 714. White bars indicate 50 µm.

Supplementary References

1. Lu, Z., Jenkyns, H. C. & Rickaby, R. E. M. Iodine to calcium ratios in marine carbonate as a paleo-redox proxy during oceanic anoxic events. *Geology* **38,** 1107–1110 (2010).
2. Davis, C. V., Wishner, K., Renema, W. & Hull, P. M. *Vertical distribution of planktic foraminifera through an Oxygen Minimum Zone:how assemblages and shell morphology reflect oxygen concentrations*. (Paleobiogeoscience: Marine Record, 2021). doi:10.5194/bg-2020-280
3. Rue, E. L., Smith, G. J., Cutter, G. A. & Bruland, K. W. The response of trace element redox couples to suboxic conditions in the water column. *Deep Sea Research Part I: Oceanographic Research Papers* **44,** 113–134 (1997).
4. Wong, G. T. F., Piumsomboon, A. U. & Dunstan, W. M. The transformation of iodate to iodide in marine phytoplankton cultures. *Marine Ecology Progress Series* **237,** 27–39 (2002).
5. Lu, Z. *et al.* Oxygen depletion recorded in upper waters of the glacial Southern Ocean. *Nat Commun* **7,** 11146 (2016).
6. Lu, W. *et al.* Refining the planktic foraminiferal I/Ca proxy: Results from the Southeast Atlantic Ocean. *Geochimica et Cosmochimica Acta* **287,** 318–327 (2020).
7. Hess, A. V., Rosenthal, Y., Zhou, X. & Bu, K. The I/Ca paleo-oxygenation proxy in planktonic foraminifera: A multispecies core-top calibration. *Geochimica et Cosmochimica Acta* (in press). doi:https://doi.org/10.1016/j.gca.2025.01.018
8. Betzler, C. *et al.* The abrupt onset of the modern South Asian Monsoon winds. *Sci Rep* **6,** 29838 (2016).
9. Bialik, O. M. *et al.* Monsoons, Upwelling, and the Deoxygenation of the Northwestern Indian Ocean in Response to Middle to Late Miocene Global Climatic Shifts. *Paleoceanography and Paleoclimatology* **35,** (2020).
10. Calvert, S. E. & Pedersen, T. F. Sedimentary geochemistry of manganese; implications for the environment of formation of manganiferous black shales. *Economic Geology* **91,** 36–47 (1996).
11. Calvert, S. E. & Pedersen, T. F. Geochemistry of Recent oxic and anoxic marine sediments: Implications for the geological record. *Marine Geology* **113,** 67–88 (1993).
12. Boyle, E. A. Manganese carbonate overgrowths on foraminifera tests. *Geochimica et Cosmochimica Acta* **47,** 1815–1819 (1983).
13. Cline, J. D. & Kaplan, I. R. Isotopic fractionation of dissolved nitrate during denitrification in the eastern tropical north pacific ocean. *Marine Chemistry* **3,** 271–299 (1975).
14. Brandes, J. A. & Devol, A. H. A global marine-fixed nitrogen isotopic budget: Implications for Holocene nitrogen cycling. *Global Biogeochemical Cycles* **16,** 67-1-67–14 (2002).
15. Sigman, D. M. & Fripiat, F. in *Encyclopedia of Ocean Sciences* 263–278 (Elsevier, 2019). doi:10.1016/B978-0-12-409548-9.11605-7
16. Kast, E. R. *et al.* Nitrogen isotope evidence for expanded ocean suboxia in the early Cenozoic. *Science* **364,** 386–389 (2019).
17. Auderset, A. *et al.* Enhanced ocean oxygenation during Cenozoic warm periods. *Nature* **609,** 77–82 (2022).
18. Hess, A. V. *et al.* A well-oxygenated eastern tropical Pacific during the warm Miocene. *Nature* **619,** 521–525 (2023).
19. Moretti, S. *et al.* Oxygen rise in the tropical upper ocean during the Paleocene-Eocene Thermal Maximum. *Science* **383,** 727–731 (2024).
20. Martinez-Garcia, A. *et al.* Laboratory assessment of the impact of chemical oxidation, mineral dissolution, and heating on the nitrogen isotopic composition of fossil-bound organic matter. *Geochemistry, Geophysics, Geosystems* (2022). doi:10.1002/essoar.10510728.1
21. Brandes, J. A. & Devol, A. H. Isotopic fractionation of oxygen and nitrogen in coastal marine sediments. *Geochimica et Cosmochimica Acta* **61,** 1793–1801 (1997).

22. Knapp, A. N., Sigman, D. M. & Lipschultz, F. N isotopic composition of dissolved organic nitrogen and nitrate at the Bermuda Atlantic Time-series Study site. *Global Biogeochemical Cycles* **19,** (2005).
23. Pena, L. D., Calvo, E., Cacho, I., Eggins, S. & Pelejero, C. Identification and removal of Mn-Mg-rich contaminant phases on foraminiferal tests: Implications for Mg/Ca past temperature reconstructions. *Geochemistry, Geophysics, Geosystems* **6,** (2005).
24. Pena, L. D. *et al.* Characterization of contaminant phases in foraminifera carbonates by electron microprobe mapping. *Geochemistry, Geophysics, Geosystems* **9,** (2008).
25. Hardisty, D. S. *et al.* Perspectives on Proterozoic surface ocean redox from iodine contents in ancient and recent carbonate. *Earth and Planetary Science Letters* **463,** 159–170 (2017).
26. Zhou, X., Thomas, E., Rickaby, R. E. M., Winguth, A. M. E. & Lu, Z. I/Ca evidence for upper ocean deoxygenation during the PETM. *Paleoceanography* **29,** 964–975 (2014).
27. Zhou, X., Hess, A. V., Bu, K., Sagawa, T. & Rosenthal, Y. Simultaneous determination of I/Ca and other elemental ratios in foraminifera using sector field ICP-MS. *Geochemistry, Geophysics, Geosystems* **23,** e2022GC010660 (2022).
28. Baker, P. A., Gieskes, J. M. & Elderfield, H. Diagenesis of Carbonates in Deep-Sea Sediments--Evidence From SR/CA Ratios and Interstitial Dissolved SR2+ Data. (1982).
29. Sosdian, S. M. *et al.* Constraining the evolution of Neogene ocean carbonate chemistry using the boron isotope pH proxy. *Earth and Planetary Science Letters* **498,** 362–376 (2018).
30. Davis, C. V., Fehrenbacher, J. S., Benitez-Nelson, C. & Thunell, R. C. Trace Element Heterogeneity Across Individual Planktic Foraminifera from the Modern Cariaco Basin. *Journal of Foraminiferal Research* **50,** 204–218 (2020).
31. Davis, C. V., Doherty, S., Fehrenbacher, J. & Wishner, K. Trace element composition of modern planktic foraminifera from an oxygen minimum zone: Potential proxies for an enigmatic environment. *Frontiers in Marine Science* **10,** (2023).
32. Yu, J., Elderfield, H., Greaves, M. & Day, J. Preferential dissolution of benthic foraminiferal calcite during laboratory reductive cleaning. *Geochemistry, Geophysics, Geosystems* **8,** (2007).
33. Klinkhammer, G. P. & Bender, M. L. The distribution of manganese in the Pacific Ocean. *Earth and Planetary Science Letters* **46,** 361–384 (1980).
34. Hardisty, D. S. *et al.* Limited iodate reduction in shipboard seawater incubations from the Eastern Tropical North Pacific oxygen deficient zone. *Earth and Planetary Science Letters* **554,** 116676 (2021).
35. Altabet, M. A., Francois, R., Murray, D. W. & Prell, W. L. Climate-related variations in denitrification in the Arabian Sea from sediment 15N/14N ratios. *Nature* **373,** 506–509 (1995).
36. Altabet, M. A., Murray, D. W. & Prell, W. L. Climatically linked oscillations in Arabian Sea denitrification over the past 1 m.y.: Implications for the marine N cycle. *Paleoceanography* **14,** 732–743 (1999).
37. Gaye-Haake, B. *et al.* Stable nitrogen isotopic ratios of sinking particles and sediments from the northern Indian Ocean. *Marine Chemistry* **96,** 243–255 (2005).
38. Tripathi, S., Tiwari, M., Lee, J. & Khim, B.-K. First evidence of denitrification vis-à-vis monsoon in the Arabian Sea since Late Miocene. *Sci Rep* **7,** 43056 (2017).
39. Ren, H., Sigman, D. M., Thunell, R. C. & Prokopenko, M. G. Nitrogen isotopic composition of planktonic foraminifera from the modern ocean and recent sediments. *Limnol. Oceanogr.* **57,** 1011–1024 (2012).
40. Smart, S. M. *et al.* Ground-truthing the planktic foraminifer-bound nitrogen isotope paleo-proxy in the Sargasso Sea. *Geochimica et Cosmochimica Acta* **235,** 463–482 (2018).
41. Rafter, P. A., Bagnell, A., Marconi, D. & DeVries, T. Global trends in marine nitrate N isotopes from observations and a neural network-based climatology. *Biogeosciences* **16,** 2617–2633 (2019).
42. Locarnini, M. M. *et al.* World ocean atlas 2018, volume 1: Temperature. (2018).
43. Garcia, H. E. *et al.* World ocean atlans 2018, volume 3: Dissolved oxygen, apparent oxygen utilization, and dissolved oxygen saturation. (2019).

44. Fripiat, F. *et al.* Nitrogen isotopic constraints on nutrient transport to the upper ocean. *Nat. Geosci.* **14,** 855–861 (2021).
45. Schlitzer, R. Ocean Data View. (2023). at <https://odv.awi.de>
46. Lougheed, B. C. & Obrochta, S. P. A Rapid, Deterministic Age-Depth Modeling Routine for Geological Sequences With Inherent Depth Uncertainty. *Paleoceanography and Paleoclimatology* **34,** 122–133 (2019).
47. *Geologic Time Scale 2020*. **2,** (Elsevier BV, 2020).
48. Shipboard Scientific Party. in *Proceedings of the Ocean Drilling Program* **117,** 555–584 (Ocean Drilling Program, 1989).
49. Shipboard Scientific Party. in *Proceedings of the Ocean Drilling Program* **115,** 847–915 (1988).
50. Pagani, M., Arthur, M. A. & Freeman, K. H. Miocene evolution of atmospheric carbon dioxide. *Paleoceanography* **14,** 273–292 (1999).
51. Inglis, G. N. *et al.* Descent toward the Icehouse: Eocene sea surface cooling inferred from GDGT distributions. *Paleoceanography* **30,** 1000–1020 (2015).
52. Westerhold, T. *et al.* An astronomically dated record of Earth's climate and its predictability over the last 66 million years | Science. (2020). at <https://www.science.org/doi/full/10.1126/science.aba6853>
53. Wang, J. *et al.* C4 expansion of Central Asia in the middle Miocene linked to the strengthening Indian monsoon. *Global and Planetary Change* 104096 (2023). doi:10.1016/j.gloplacha.2023.104096
54. Auderset, A. *et al.* Sea Level Modulation of Atlantic Nitrogen Fixation Over Glacial Cycles. *Paleoceanography and Paleoclimatology* **39,** e2024PA004878 (2024).
55. Zhuang, G., Pagani, M. & Zhang, Y. G. Monsoonal upwelling in the western Arabian Sea since the middle Miocene. *Geology* **45,** 655–658 (2017).
56. Yang, X., Groeneveld, J., Jian, Z., Steinke, S. & Giosan, L. Middle Miocene Intensification of South Asian Monsoonal Rainfall. *Paleoceanography and Paleoclimatology* **35,** e2020PA003853 (2020).
57. Auer, G., Bialik, O. M., Antoulas, M.-E., Vogt-Vincent, N. & Piller, W. E. Biotic response of plankton communities to Middle to Late Miocene monsoon wind and nutrient flux changes in the Oman margin upwelling zone. *Climate of the Past* **19,** 2313–2340 (2023).
58. Gupta, A. K., Yuvaraja, A., Prakasam, M., Clemens, S. C. & Velu, A. Evolution of the South Asian monsoon wind system since the late Middle Miocene. *Palaeogeography, Palaeoclimatology, Palaeoecology* **438,** 160–167 (2015).
59. Beasley, C. *et al.* Evidence of a South Asian Proto-Monsoon During the Oligocene-Miocene Transition. *Paleoceanography and Paleoclimatology* **36,** e2021PA004278 (2021).
60. Clift, P. D. *et al.* Correlation of Himalayan exhumation rates and Asian monsoon intensity. *Nature Geosci* **1,** 875–880 (2008).
61. Guo, Z. T. *et al.* Onset of Asian desertification by 22 Myr ago inferred from loess deposits in China. *Nature* **416,** 159–163 (2002).
62. Smart, C. W. & Ramsay, A. T. S. Benthic foraminiferal evidence for the existence of an early Miocene oxygen-depleted oceanic water mass? *Journal of the Geological Society* **152,** 735–738 (1995).
63. Wright, J. D., Miller, K. G. & Fairbanks, R. G. Early and Middle Miocene stable isotopes: Implications for Deepwater circulation and climate. *Paleoceanography* **7,** 357–389 (1992).
64. Flower, B. P. & Kennett, J. P. Middle Miocene deepwater paleoceanography in the southwest Pacific: Relations with East Antarctic Ice Sheet development. *Paleoceanography* **10,** 1095–1112 (1995).
65. Woodruff, F. & Savin, S. M. Miocene deepwater oceanography. *Paleoceanography* **4,** 87–140 (1989).
66. Gourlan, A. T., Meynadier, L. & Allègre, C. J. Tectonically driven changes in the Indian Ocean circulation over the last 25 Ma: Neodymium isotope evidence. *Earth and Planetary Science Letters* **267,** 353–364 (2008).
