# Supplementary material for: Contrasting evolution of the Arabian Sea and Pacific Ocean oxygen minimum zones during the Miocene
Source: Commun Earth Environ. 2026 Jan 16;7(1):47. doi: 10.1038/s43247-025-03112-4 (PMC12811131; doi:10.1038/s43247-025-03112-4)
Supplement: Supplementary file 3 — Description of Additional Supplementary File [file 43247_2025_3112_MOESM3_ESM.pdf]

Description of Additional Supplementary Files

File name: Supplementary Data 1

Description: Age-depth correlation for Site 714 based on biostratigraphic occurrence data and for Site 730 based on biostratigraphic occurrence data and strontium isotope chemostratigraphy. Updated age models for Sites 722, 754, 761 and NGHP-01-01A, used for temperature compilation.

File name: Supplementary Data 2

Description: Trace element data for Sites 714 and 730 (Mg/Ca, I/Ca, Mn/Ca, Sr/Ca, Fe/Ca) and reconstructed temperatures based on Mg/Ca.

File name: Supplementary Data 3

Description: Foraminifera-bound nitrogen isotope data for Sites 714 and 730 and bulk sedimentary nitrogen isotope data for Site 714.

File name: Supplementary Data 4

Description: GDGT data for Site 730, including concentrations for isoprenoid GDGTs, branched GDGTs and Crenaracheol, as well as calculated “Red Sea-type” index.
