# Supplementary material for: Contrasting evolution of the Arabian Sea and Pacific Ocean oxygen minimum zones during the Miocene
Source: Commun Earth Environ. 2026 Jan 16;7(1):47. doi: 10.1038/s43247-025-03112-4 (PMC12811131; doi:10.1038/s43247-025-03112-4)
Supplement: Supplementary file 8 — Reporting Summary [file 43247_2025_3112_MOESM8_ESM.pdf]

nature portfolio

Corresponding author(s): COMMSENV-25-3636-T

Last updated by author(s): Nov 12, 2025

Reporting Summary

Nature Portfolio wishes to improve the reproducibility of the work that we publish. This form provides structure for consistency and transparency in reporting. For further information on Nature Portfolio policies, see our Editorial Policies and the Editorial Policy Checklist.

Statistics

For all statistical analyses, confirm that the following items are present in the figure legend, table legend, main text, or Methods section.

n/a	Confirmed	
☒	☐	The exact sample size (n) for each experimental group/condition, given as a discrete number and unit of measurement
☐	☒	A statement on whether measurements were taken from distinct samples or whether the same sample was measured repeatedly
☒	☐	The statistical test(s) used AND whether they are one- or two-sided<br>*Only common tests should be described solely by name; describe more complex techniques in the Methods section.*
☒	☐	A description of all covariates tested
☒	☐	A description of any assumptions or corrections, such as tests of normality and adjustment for multiple comparisons
☐	☒	A full description of the statistical parameters including central tendency (e.g. means) or other basic estimates (e.g. regression coefficient) AND variation (e.g. standard deviation) or associated estimates of uncertainty (e.g. confidence intervals)
☒	☐	For null hypothesis testing, the test statistic (e.g. F, t, r) with confidence intervals, effect sizes, degrees of freedom and P value noted<br>*Give P values as exact values whenever suitable.*
☒	☐	For Bayesian analysis, information on the choice of priors and Markov chain Monte Carlo settings
☒	☐	For hierarchical and complex designs, identification of the appropriate level for tests and full reporting of outcomes
☒	☐	Estimates of effect sizes (e.g. Cohen's d, Pearson's r), indicating how they were calculated

Our web collection on statistics for biologists contains articles on many of the points above.

Software and code

Policy information about availability of computer code

Data collection	n/a
Data analysis	Microsoft office, Ocean Data View (Schlitzer, R. Ocean Data View. (2023). at <https://odv.awi.de>), Undatable (Lougheed, B. C. & Obrochta, S. P. A Rapid, Deterministic Age-Depth Modeling Routine for Geological Sequences With Inherent Depth Uncertainty. Paleoceanography and Paleoclimatology 34, 122–133 (2019)).

For manuscripts utilizing custom algorithms or software that are central to the research but not yet described in published literature, software must be made available to editors and reviewers. We strongly encourage code deposition in a community repository (e.g. GitHub). See the Nature Portfolio guidelines for submitting code & software for further information.

Data

Policy information about availability of data

All manuscripts must include a data availability statement. This statement should provide the following information, where applicable:

- Accession codes, unique identifiers, or web links for publicly available datasets
- A description of any restrictions on data availability
- For clinical datasets or third party data, please ensure that the statement adheres to our policy

All data are available at PANGAEA https://doi.org/10.1594/PANGAEA.982881. Data_S1_age_model.xlsx contains updated age models to GTS20 for ODP Sites 730,

714, 761, 754, 722, and NGHP-01-01A. Data_S2_trace_element.xlsx contains trace element data for ODP Sites 730 and 714. Data_S3_nitrogen_isotopes.xlsx contains foraminifera-bound nitrogen isotope data for ODP Sites 730 and 714. Data_S4_biomarker.xlsx contains GDGT data for ODP Site 730.

Research involving human participants, their data, or biological material

Policy information about studies with human participants or human data. See also policy information about sex, gender (identity/presentation), and sexual orientation and race, ethnicity and racism.

Reporting on sex and gender	n/a
Reporting on race, ethnicity, or other socially relevant groupings	n/a
Population characteristics	n/a
Recruitment	n/a
Ethics oversight	n/a

Note that full information on the approval of the study protocol must also be provided in the manuscript.

Field-specific reporting

Please select the one below that is the best fit for your research. If you are not sure, read the appropriate sections before making your selection.

☐ Life sciences ☐ Behavioural & social sciences ☒ Ecological, evolutionary & environmental sciences

For a reference copy of the document with all sections, see nature.com/documents/nr-reporting-summary-flat.pdf

Ecological, evolutionary & environmental sciences study design

All studies must disclose on these points even when the disclosure is negative.

Study description	We measured trace elements and isotopes in planktonic foraminifera (microfossils) from deep-sea sediment cores collected by the international ocean discovery program (IODP). Samples were chosen to give sufficient temporal resolution for the multi-million year time span in question.
Research sample	We use trace elements and isotopes in planktonic foraminifera fossils. These organisms live in the upper water column and can tell us about its properties.
Sampling strategy	The number of samples is limited by the amount and size of foraminifera present in the sediments. Since these come from deep-sea sediments, samples are extremely precious and larger samples is not possible.
Data collection	Data are measured using instrumentation at Rutgers University and Max Planck Institute for Chemistry.
Timing and spatial scale	This is a study of past ocean oxygenation, investigating a key warm interval ca. 16 Million years ago.
Data exclusions	Rare outliers are excluded, generally >2 stdev from the mean of the data from a time period, which indicates a contaminated sample.
Reproducibility	The number of foraminifera available determines the number of replicates. For example, for nitrigen isotopes, ~400 foraminifera are needed and no replicates are possible. Instead we used an internal foraminifera standard for reproducibility. For trace elements, 13 replicates were possible.
Randomization	Samples were randomized at different levels. Some of them were run in separate runs.
Blinding	We did not blind our samples, they were labeled with a sample ID, which was only meaningful with the sample list stored separately. We cannot influence the geochemical characteristics of the fossils, so blinding is not typically performed in our field.

Did the study involve field work? ☐ Yes ☒ No

Reporting for specific materials, systems and methods

We require information from authors about some types of materials, experimental systems and methods used in many studies. Here, indicate whether each material, system or method listed is relevant to your study. If you are not sure if a list item applies to your research, read the appropriate section before selecting a response.

Materials & experimental systems

n/a	Involved in the study
☒	☐ Antibodies
☒	☐ Eukaryotic cell lines
☐	☒ Palaeontology and archaeology
☒	☐ Animals and other organisms
☒	☐ Clinical data
☒	☐ Dual use research of concern
☒	☐ Plants

Methods

n/a	Involved in the study
☒	☐ ChIP-seq
☒	☐ Flow cytometry
☒	☐ MRI-based neuroimaging

Palaeontology and Archaeology

Specimen provenance	The samples come from the international ocean discovery program; we did not collect them ourselves.
Specimen deposition	IODP manages samples
Dating methods	No new dates were measured.

☒ Tick this box to confirm that the raw and calibrated dates are available in the paper or in Supplementary Information.

Ethics oversight	no ethical approval is done in our field.

Note that full information on the approval of the study protocol must also be provided in the manuscript.

Plants

Seed stocks	n/a
Novel plant genotypes	n/a
Authentication	n/a
